# Synthesis, crystal structure, DFT calculations and Hirshfeld surface analysis of 2-(1-decyl-2-oxo­indolin-3-yl­idene)propanedi­nitrile

**DOI:** 10.1107/S2056989018017267

**Published:** 2019-01-01

**Authors:** Ibtissam Rayni, Youness El Bakri, Chin-Hung Lai, L’houssaine El Ghayati, El Mokhtar Essassi, Joel T. Mague

**Affiliations:** a Laboratoire de Chimie Organique Hétérocyclique, Centre de Recherche des Sciences des Médicaments, URAC 21, Pôle de Compétence Pharmacochimie, Av Ibn Battouta, BP 1014, Faculté des Sciences, Université Mohammed V, Rabat, Morocco; b Organic Chemistry Department, Science Faculty, RUDN University, Miklukho-Maklaya st. 6, 117198 Moscow, Russian Federation; c Department of Medical Applied Chemistry, Chung Shan Medical University, Taichung 40241, Taiwan; d Department of Medical Education, Chung Shan Medical University Hospital, 402 Taichung, Taiwan; e Department of Chemistry, Tulane University, New Orleans, LA 70118, USA

**Keywords:** crystal structure, π-stacking, indole, Hirshfeld surface analysis

## Abstract

In the title mol­ecule, C_21_H_25_N_3_O, the 1-decyl substituents are in an extended conformation and inter­calate in the crystal packing to form hydro­phobic bands. The packing is further organized by π–π-stacking inter­actions between pyrrole and phenyl rings [centroid–centroid distance = 3.6178 (11) Å] and a C=O⋯π(pyrrole) inter­action [3.447 (2) Å]. Hirshfeld surface analysis indicates that the H⋯N/N⋯H inter­actions make the highest contribution (17.4%) to the crystal packing.

## Abstract 2

In the crystal, the 1-decyl chains are in a ‘fully extended’ conformation and inter­calate in the crystal packing to form hydro­phobic bands. The packing is further organized by π–π-stacking and C=O⋯π-ring inter­actions. The inter­molecular inter­actions were investigating by Hirshfeld surface analysis.

## Chemical context   

Knoevenagel condensation is a nucleophilic addition of an active hydrogen compound to a carbonyl group followed by dehydration in which a mol­ecule of water is eliminated (Jones, 1967 ▸). The indole scaffold including isatin (1*H*-indole-2,3-dione) represents an important structural subunit for the discovery of new drug candidates (Pandeya *et al.*, 2005 ▸). The carbonyl group in the 3-position of isatin is known to be active in various condensation reactions and thus the most common methods for the synthesis of 2-(2-oxoindolin-3-yl­idene)malono­nitriles are the condensation of isatins with malono­nitrile in the presence of a catalyst, such as piperidine acetate (Kayukov *et al.*, 2011 ▸), DBU, Al_2_O_3_, N(CH_2_CH_2_OH)_3_ or chitosan (Abdelhamid, 2009 ▸). Over the past few years, mol­ecular iodine has emerged as powerful catalyst in various organic transformations (Kidwai *et al.*, 2007 ▸). As well as having the advantage of being inexpensive, non-toxic, and nature friendly, iodine affords the desired products in good to excellent yields with high selectivity.
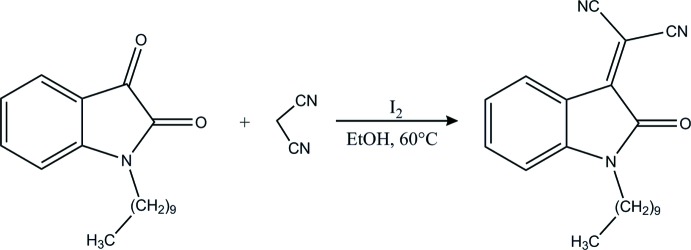


As a continuation of our research on the synthesis, functionilization, physico-chemical and biological properties of indole derivatives (Al Mamari *et al.*, 2012*a* ▸,*b* ▸,*c* ▸,*d* ▸; Rayni *et al.*, 2017 ▸, 2017*a* ▸,*b* ▸; Zarrok *et al.*, 2012 ▸), we report our results on the Knoevenagel condensation of 1-decyl­indoline-2,3-dione with malono­nitrile using mol­ecular iodine as catalyst.
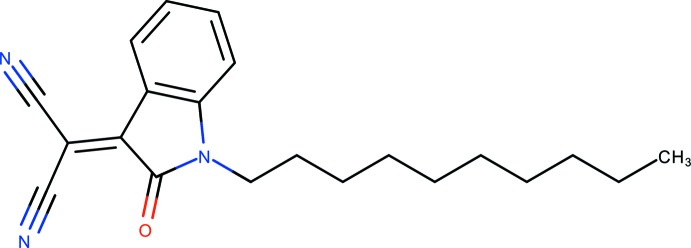


## Structural commentary   

**Figure 1:**
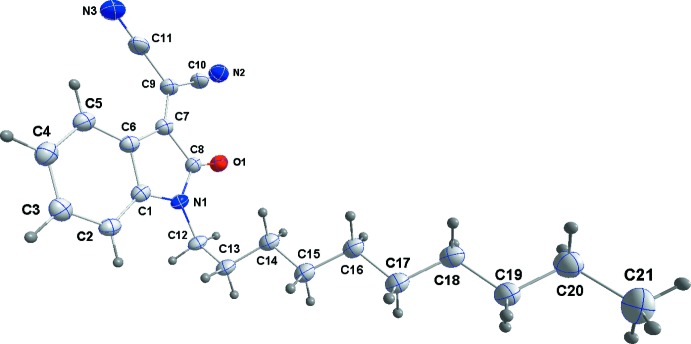
The title mol­ecule with the labelling scheme and 50% probability ellipsoids.

The 1-decyl substituent in the title compound (Fig. 1 ▸) is fully extended in the crystal and the head end is nearly perpendic­ular to the plane of the five-membered ring as shown by the C8—N1—C12—C13 torsion angle of 112.3 (2)°. The indole portion is not quite planar, as indicated by the dihedral angle of 1.64 (10)° between the constituent rings and the r.m.s. deviation of 0.015 Å. As expected, the propanedi­nitrile group is essentially coplanar with the five-membered ring, the C8—C7—C9—C11 torsion angle being 179.71 (17)°.

## Supra­molecular features   

**Figure 2:**
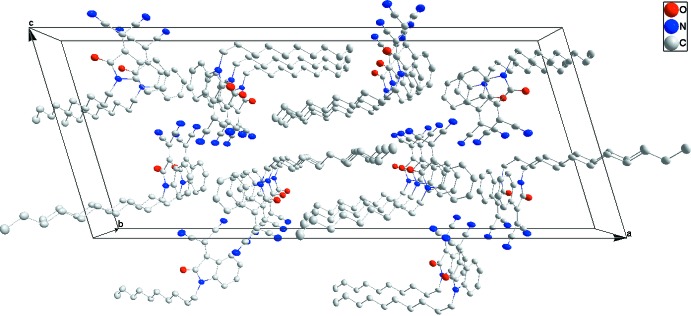
The packing viewed along the *b* axis.

**Figure 3:**
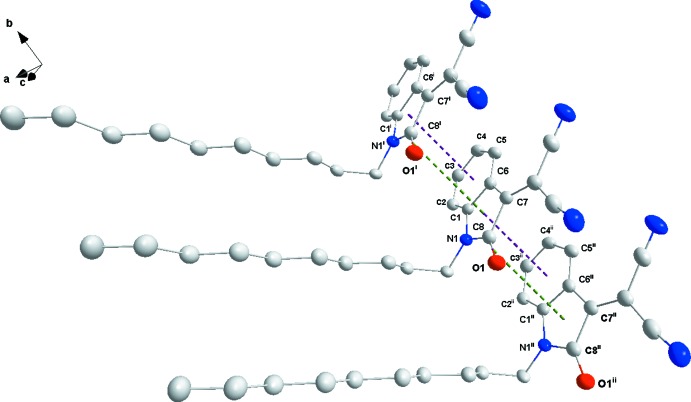
Detail of the offset π–π-stacking (purple dotted lines) and C=O⋯π(ring) (green dotted lines) inter­actions [symmetry codes: (i) *x*, −1 + *y*, *z*; (ii) *x*, 1 + *y*, *z*].

The mol­ecules pack with the 1-decyl chains inter­calating to form large hydro­phobic bands (Fig. 2 ▸) approximately parallel to the *b*-axis direction. The indole portion participates in offset π–π-stacking inter­actions in the *b*-axis direction between the five-membered ring in one mol­ecule and the six-membered ring in the next (Fig. 3 ▸) with a centroid–centroid distance of 3.6178 (11) Å and a dihedral angle of 1.64 (10)°. Reinforcing this is a C=O⋯π(ring) inter­action between C8=O1 and the five-membered ring in the adjacent mol­ecule along the *b*-axis direction (Fig. 3 ▸) with a C⋯centroid distance of 3.447 (2) Å.

## Database survey   

**Figure 4:**
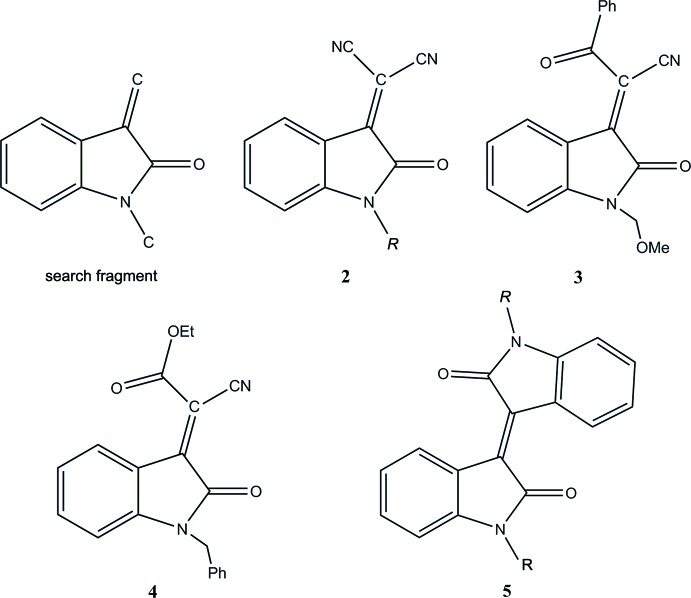
Search fragment and related compounds.

A search of the Cambridge Structural Database (Version 5.39 with updates through May 2018; Groom *et al.*, 2016 ▸) with the fragment shown in Fig. 4 ▸ yielded 133 hits of which 34 are close to the title compound in that the substituents on the methyl­idene carbon are relatively small in size. The closest analogues are **2** [*R* = CH_3_ (Wang *et al.*, 2013 ▸); (CH_2_)_5_CH_3_ (Rayni *et al.*, 2017*b* ▸)], **3** (Hu *et al.*, 2014 ▸) and **4** (Lian *et al.*, 2012 ▸) although there are also some inter­esting related compounds such as **5** [*R* = (CH_2_)_5_CH_3_; Hasegawa *et al.*, 2015 ▸), (CH_2_)_9_CH_3_ (Bogdanov *et al.*, 2014 ▸) and (CH_2_)_3_CH_3_ (Yuan & Fang, 2011 ▸), (CH_2_)_6_Br Bogdanov *et al.*, 2013 ▸)]. In these, the indole fragment varies from being planar to having a dihedral angle between the two constituent rings of up to 3.30°. The substituent on the ring nitro­gen atom is generally in an extended conformation with the head end nearly perpendicular to the plane of the five-membered ring with torsion angles corresponding to the C8—N1—C12—C13 torsion angle in the title compound varying from 73.4–104.8°.

## DFT optimization   

The structure in the gas phase of the title compound was optimized by means of density functional theory (DFT). The DFT calculation was performed by the hybrid B3LYP method, which is based on the idea of Becke and considers a mixture of the exact (Hartree–Fock) and DFT exchange utilizing the B3 functional, together with the LYP correlation functional (Becke, 1993 ▸; Lee *et al.*, 1988 ▸; Miehlich *et al.*, 1989 ▸). The B3LYP calculation was performed in conjunction with the basis set DZVP (Godbout *et al.*, 1992 ▸). It is noteworthy to mention that the double-ξ basis set used was designed for a DFT calculation. After obtaining the converged geometry, the harmonic vibrational frequencies were calculated at the same theoretical level to confirm that the number of imaginary frequencies is zero for the stationary point. Both the geometry optimization and harmonic vibrational frequency analysis of the title compound were performed with the *Gaussian16* program (Frisch *et al.*, 2016 ▸).

**Figure 5:**
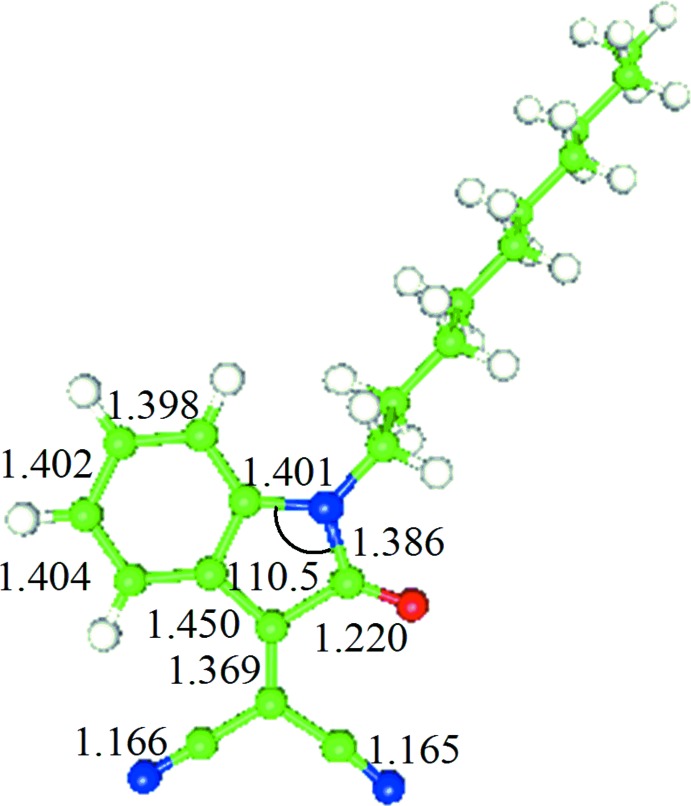
The B3LYP-optimized geometry of the title compound (Å, °).

The result of the B3LYP geometry optimization for the title compound was compared with that determined in the crystallographic study. The B3LYP-optimized geometry of the title compound is shown in Fig. 5 ▸ with selected geometric parameters of the gas-phase and the solid-phase structures summarized in Table 1 ▸. These show that the gas-phase structure shows a small deviation from the solid-phase one (Reichman *et al.*, 1969 ▸; Liao & Zhang, 1998 ▸).

## Hirshfeld surface calculations   

Both the definition of a mol­ecule in a condensed phase and the recognition of distinct entities in mol­ecular liquids and crystals are fundamental concepts in chemistry. Based on Hirshfeld’s partitioning scheme, a method was proposed to divide the electron distribution in a crystalline phase into mol­ecular fragments (Spackman & Byrom, 1997 ▸; McKinnon *et al.*, 2004 ▸; Spackman & Jayatilaka, 2009 ▸). This method partitioned the crystal into regions where the electron distribution of a sum of spherical atoms for the mol­ecule dominates over the corresponding sum of the crystal. As it is derived from Hirshfeld’s stockholder partitioning, the mol­ecular surface is named as the Hirshfeld surface. In this study, the Hirshfeld surface analysis of the title compound was performed utilizing the *CrystalExplorer* program (Turner *et al.*, 2017 ▸).

**Figure 6:**
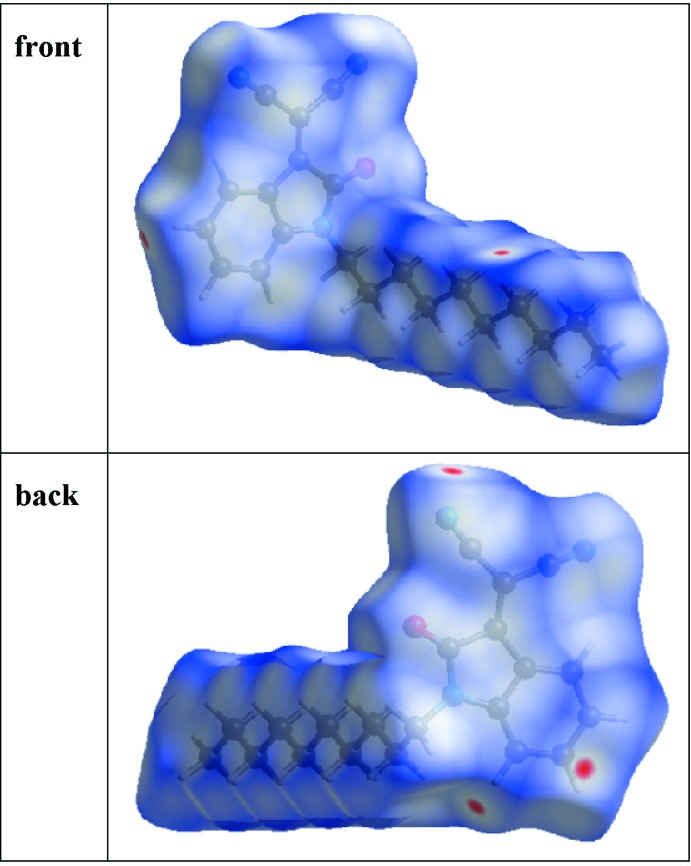
The *d*_norm_ Hirshfeld surface of the title compound (red: negative, white: zero, blue: positive; scale: −0.0774 to 1.3395 a.u.).

The standard resolution mol­ecular Hirshfeld surface (*d*_norm_) of the title compound is shown in Fig. 6 ▸. The 3D *d*_norm_ surface is used to identify close inter­molecular inter­actions. The value of *d*_norm_ is negative (positive) when inter­molecular contacts are shorter (longer) than the van der Waals radii. The *d*_norm_ value is mapped onto the Hirshfeld surface using red, white and blue. The red regions represent closer contacts with a negative *d*_norm_ value while the blue regions represent longer contacts with a positive *d*_norm_ value and the white regions represent contacts equal to the van der Waals separation and have a *d*_norm_ value of zero. As shown in Fig. 6 ▸, the major inter­actions in the title compound are inter­molecular H⋯O and H⋯N hydrogen bonds.

**Figure 7:**
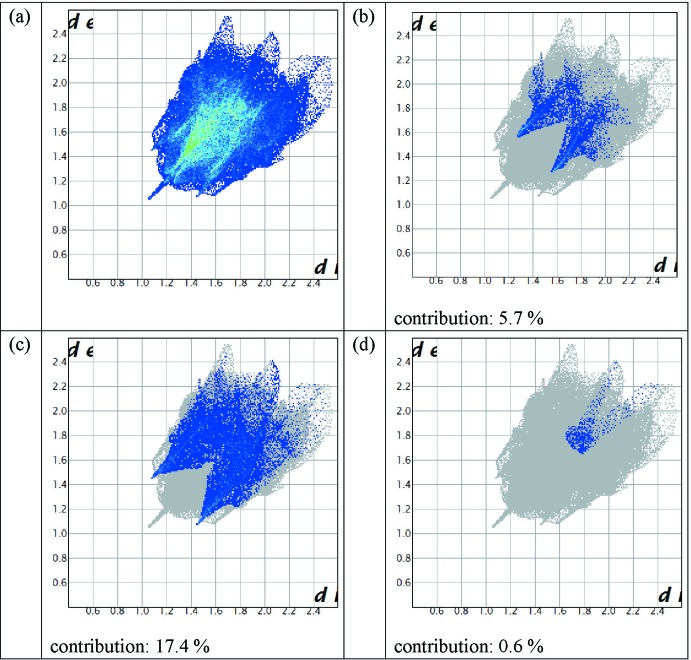
The two-dimensional fingerprint plot of the title compound (*a*) full and decomposed into (*b*) H⋯O/O⋯H contacts, (*c*) H⋯N/N⋯H contacts and (*d*) N⋯N contacts.

The 2D fingerprint plots highlight particular atom-pair contacts and enable the separation of contributions from different inter­action types that overlap in the full fingerprint. Using the standard 0.6–2.6 view with the *d*_e_ and *d*_i_ distance scales displayed on the graph axes and including the reciprocal contacts, the contribution of the H⋯N contacts is larger than that of the H⋯O contacts (Fig. 7 ▸). Inter­estingly, we found that there is a negligible contribution of N⋯N contacts (Govers, 1975 ▸; Cartwright & Wilkinson, 2010 ▸). This inter­action might be considered as a stabilizing hyperconjugative one between a π-bonding orbital of one C≡N group and a π*-bonding orbital of another [C≡N group π(CN) → π*(C′N′); Jeong & Kwon, 2000 ▸].

## Synthesis and crystallization   

A mixture of 1-decyl­indole-2,3-dione (0,5g, 2.1 mmol), malono­nitrile (0,14g, 2.1 mmol), and I_2_ (0.05g, 0.21 mmol) in ethanol (10 mL) was heated at 333 K. After completion of the reaction (monitored by TLC), the mixture was treated with aqueous Na_2_S_2_O_3_ solution and extracted with ethyl acetate (2 × 10 mL). The extract was dried over sodium sulfate, filtered and the solvent was evaporated *in vacuo*. The purified product was recrystallized from ethanol solution to afford the title compound as orange, plate-like crystals.

## Refinement   

Crystal data, data collection and structure refinement details are summarized in Table 2 ▸. Trial refinements of the model with the one-component reflection file extracted from the full twinned data with *TWINABS* and with the full, two-component reflection file indicated that the former gave better results both in terms of lower values of *R*_1_ and *wR*_2_ and in lower s.u. values for derived parameters.

## Supplementary Material

Crystal structure: contains datablock(s) global, I. DOI: 10.1107/S2056989018017267/vm2214sup1.cif

Structure factors: contains datablock(s) I. DOI: 10.1107/S2056989018017267/vm2214Isup2.hkl

Supporting information file. DOI: 10.1107/S2056989018017267/vm2214Isup3.cdx

Supporting information file. DOI: 10.1107/S2056989018017267/vm2214Isup4.cml

CCDC reference: 1883193

Additional supporting information:  crystallographic information; 3D view; checkCIF report

## Figures and Tables

**Table 1:** The B3LYP-optimized and X-ray structural parameters for **1** (Å, °)

	B3LYP	X-ray
C1—C6	1.421	1.410 (2)
C6—C5	1.391	1.393 (3)
C5—C4	1.404	1.389 (3)
C4—C3	1.402	1.386 (3)
C3—C2	1.398	1.396 (3)
C2—C1	1.402	1.377 (3)
C1—N1	1.401	1.406 (2)
N1—C8	1.386	1.372 (2)
C8—O1	1.220	1.214 (2)
C8—C7	1.522	1.520 (2)
C7—C6	1.450	1.440 (2)
C7—C9	1.396	1.350 (3)
C9—C10	1.437	1.437 (3)
C9—C11	1.436	1.444 (3)
C10—N2	1.165	1.147 (3)
C11—N3	1.166	1.142 (3)
N1—C12	1.461	1.461 (2)
C12—C13	1.536	1.526 (2)
		
C7—C8—N1	106.1	105.90 (15)
C11—C9—C10	114.6	114.51 (16)
C8—N1—C1	110.5	110.66 (14)

**Table 2:** Experimental details

Crystal data	
Chemical formula	C_21_H_25_N_3_O
*M*_r_	335.44
Crystal system, space group	Monoclinic, *C*2/*c*
Temperature (K)	150
*a*, *b*, *c* (Å)	44.4837 (12), 4.7293 (1), 18.3432 (5)
β (°)	106.965 (2)
*V* (Å^3^)	3691.05 (17)
*Z*	8
Radiation type	Cu *K*α
μ (mm^−1^)	0.59
Crystal size (mm)	0.29 × 0.08 × 0.03

Data collection	
Diffractometer	Bruker D8 VENTURE PHOTON 100 CMOS
Absorption correction	Multi-scan (*TWINABS*; Sheldrick, 2009 ▸)
*T*_min_, *T*_max_	0.75, 0.98
No. of measured, independent and observed [*I* > 2σ(*I*)] reflections	25556, 3592, 2656
*R*_int_	0.054
(sin θ/λ)_max_ (Å^−1^)	0.618

Refinement	
*R*[*F* ^2^ > 2σ(*F* ^2^)], *wR*(*F* ^2^), *S*	0.051, 0.134, 1.05
No. of reflections	3592
No. of parameters	326
H-atom treatment	All H-atom parameters refined
Δρ_max_, Δρ_min_ (e Å^−3^)	0.24, −0.20

Computer programs: *APEX3* and *SAINT* (Bruker, 2016 ▸), *SHELXT* (Sheldrick, 2015*a* ▸), *SHELXL 2014/7* (Sheldrick, 2015*b* ▸), *DIAMOND* (Brandenburg & Putz, 2012 ▸) and *SHELXTL* (Sheldrick, 2008 ▸).

## supplementary crystallographic information

### Crystal data

**Table:**

C_21_H_25_N_3_O	*F*(000) = 1440
*M**_r_* = 335.44	*D*_x_ = 1.207 Mg m^−^^3^
Monoclinic, *C*2/*c*	Cu *K*α radiation, λ = 1.54178 Å
*a* = 44.4837 (12) Å	Cell parameters from 9886 reflections
*b* = 4.7293 (1) Å	θ = 4.2–72.3°
*c* = 18.3432 (5) Å	µ = 0.59 mm^−^^1^
β = 106.965 (2)°	*T* = 150 K
*V* = 3691.05 (17) Å^3^	Plate, orange
*Z* = 8	0.29 × 0.08 × 0.03 mm

### Data collection

**Table:**

Bruker D8 VENTURE PHOTON 100 CMOS diffractometer	3592 independent reflections
Radiation source: INCOATEC IµS micro–focus source	2656 reflections with *I* > 2σ(*I*)
Mirror monochromator	*R*_int_ = 0.054
Detector resolution: 10.4167 pixels mm^-1^	θ_max_ = 72.3°, θ_min_ = 2.1°
ω scans	*h* = −54→5
Absorption correction: multi-scan (*TWINABS*; Sheldrick, 2009)	*k* = −5→5
*T*_min_ = 0.75, *T*_max_ = 0.98	*l* = −21→22
25556 measured reflections	

### Refinement

**Table:**

Refinement on *F*^2^	Primary atom site location: structure-invariant direct methods
Least-squares matrix: full	Secondary atom site location: difference Fourier map
*R*[*F*^2^ > 2σ(*F*^2^)] = 0.051	Hydrogen site location: difference Fourier map
*wR*(*F*^2^) = 0.134	All H-atom parameters refined
*S* = 1.05	*w* = 1/[σ^2^(*F*_o_^2^) + (0.0647*P*)^2^ + 0.6236*P*] where *P* = (*F*_o_^2^ + 2*F*_c_^2^)/3
3592 reflections	(Δ/σ)_max_ = 0.001
326 parameters	Δρ_max_ = 0.24 e Å^−^^3^
0 restraints	Δρ_min_ = −0.20 e Å^−^^3^

### Special details

**Table:**

Experimental. Analysis of 1401 reflections having I/σ(I) > 15 and chosen from the full data set with *CELL_NOW* (Sheldrick, 2008) showed the crystal to belong to the triclinic system and to be twinned by a 180° rotation about the *a* axis. The raw data were processed using the multi-component version of*SAINT* under control of the two-component orientation file generated by *CELL_NOW*.
Geometry. All esds (except the esd in the dihedral angle between two l.s. planes) are estimated using the full covariance matrix. The cell esds are taken into account individually in the estimation of esds in distances, angles and torsion angles; correlations between esds in cell parameters are only used when they are defined by crystal symmetry. An approximate (isotropic) treatment of cell esds is used for estimating esds involving l.s. planes.
Refinement. Refinement of F^2^ against ALL reflections. The weighted R-factor wR and goodness of fit S are based on F^2^, conventional R-factors R are based on F, with F set to zero for negative F^2^. The threshold expression of F^2^ > 2sigma(F^2^) is used only for calculating R-factors(gt) etc. and is not relevant to the choice of reflections for refinement. R-factors based on F^2^ are statistically about twice as large as those based on F, and R- factors based on ALL data will be even larger. Trial refinements with the single-component reflection file extracted from the full data set with *TWINABS* and with the complete two-component reflection file showed the former refinement to be the better one.

### Fractional atomic coordinates and isotropic or equivalent isotropic displacement parameters (Å^2^)

**Table:**

	*x*	*y*	*z*	*U*_iso_*/*U*_eq_	
O1	0.36446 (3)	−0.1282 (3)	0.67635 (8)	0.0362 (3)	
N1	0.34709 (3)	0.1596 (3)	0.75794 (8)	0.0271 (3)	
N2	0.34275 (4)	−0.2025 (5)	0.49015 (10)	0.0470 (5)	
N3	0.27212 (4)	0.4371 (5)	0.45464 (10)	0.0495 (5)	
C1	0.32328 (4)	0.3634 (4)	0.74915 (10)	0.0262 (4)	
C2	0.31433 (4)	0.5037 (4)	0.80525 (10)	0.0286 (4)	
H2	0.3249 (5)	0.469 (5)	0.8594 (13)	0.032 (5)*	
C3	0.28988 (4)	0.6993 (4)	0.78192 (11)	0.0317 (4)	
H3	0.2834 (5)	0.807 (5)	0.8209 (13)	0.041 (6)*	
C4	0.27473 (4)	0.7480 (4)	0.70550 (11)	0.0319 (4)	
H4	0.2582 (5)	0.880 (5)	0.6893 (12)	0.034 (5)*	
C5	0.28324 (4)	0.5999 (4)	0.64921 (11)	0.0303 (4)	
H5	0.2723 (5)	0.633 (5)	0.5964 (14)	0.039 (6)*	
C6	0.30782 (4)	0.4059 (4)	0.67108 (10)	0.0267 (4)	
C7	0.32211 (4)	0.2166 (4)	0.62943 (10)	0.0273 (4)	
C8	0.34757 (4)	0.0565 (4)	0.68839 (10)	0.0283 (4)	
C9	0.31586 (4)	0.1659 (4)	0.55402 (10)	0.0301 (4)	
C10	0.33204 (4)	−0.0402 (5)	0.52187 (10)	0.0346 (4)	
C11	0.29150 (4)	0.3188 (5)	0.49872 (11)	0.0354 (4)	
C12	0.36711 (4)	0.0513 (4)	0.83047 (10)	0.0296 (4)	
H12A	0.3786 (5)	−0.129 (5)	0.8172 (12)	0.036 (6)*	
H12B	0.3527 (5)	0.000 (5)	0.8620 (12)	0.032 (5)*	
C13	0.39191 (4)	0.2618 (4)	0.87386 (10)	0.0298 (4)	
H13A	0.4035 (5)	0.161 (5)	0.9230 (13)	0.039 (6)*	
H13B	0.3805 (5)	0.432 (5)	0.8878 (12)	0.032 (5)*	
C14	0.41561 (4)	0.3492 (4)	0.83250 (11)	0.0317 (4)	
H14A	0.4257 (5)	0.170 (5)	0.8154 (13)	0.044 (6)*	
H14B	0.4049 (5)	0.453 (5)	0.7845 (13)	0.034 (5)*	
C15	0.44154 (4)	0.5345 (4)	0.88269 (11)	0.0323 (4)	
H15A	0.4527 (5)	0.429 (5)	0.9310 (14)	0.044 (6)*	
H15B	0.4321 (5)	0.709 (5)	0.9023 (12)	0.034 (5)*	
C16	0.46614 (4)	0.6289 (4)	0.84511 (11)	0.0335 (4)	
H16A	0.4555 (6)	0.739 (5)	0.7968 (14)	0.047 (6)*	
H16B	0.4759 (5)	0.461 (5)	0.8270 (12)	0.040 (6)*	
C17	0.49228 (4)	0.8068 (5)	0.89736 (11)	0.0330 (4)	
H17A	0.5025 (6)	0.692 (5)	0.9449 (15)	0.052 (7)*	
H17B	0.4824 (5)	0.979 (5)	0.9136 (13)	0.042 (6)*	
C18	0.51688 (4)	0.9018 (5)	0.85996 (11)	0.0355 (5)	
H18A	0.5257 (6)	0.730 (5)	0.8419 (14)	0.048 (6)*	
H18B	0.5063 (6)	1.017 (5)	0.8130 (15)	0.051 (7)*	
C19	0.54344 (4)	1.0786 (5)	0.91077 (11)	0.0341 (4)	
H19A	0.5547 (5)	0.968 (5)	0.9594 (14)	0.046 (6)*	
H19B	0.5334 (5)	1.249 (5)	0.9312 (13)	0.041 (6)*	
C20	0.56740 (5)	1.1722 (5)	0.87134 (13)	0.0403 (5)	
H20A	0.5768 (6)	0.999 (6)	0.8527 (14)	0.052 (7)*	
H20B	0.5552 (5)	1.280 (5)	0.8244 (14)	0.046 (6)*	
C21	0.59341 (5)	1.3554 (6)	0.92162 (16)	0.0483 (6)	
H21A	0.5840 (6)	1.529 (6)	0.9393 (15)	0.061 (8)*	
H21B	0.6081 (7)	1.418 (6)	0.8951 (17)	0.067 (8)*	
H21C	0.6055 (6)	1.242 (6)	0.9663 (17)	0.062 (8)*	

### Atomic displacement parameters (Å^2^)

**Table:**

	*U*^11^	*U*^22^	*U*^33^	*U*^12^	*U*^13^	*U*^23^
O1	0.0383 (7)	0.0343 (8)	0.0341 (7)	0.0090 (6)	0.0073 (5)	−0.0011 (6)
N1	0.0288 (7)	0.0250 (8)	0.0238 (8)	0.0017 (6)	0.0020 (5)	0.0023 (6)
N2	0.0512 (10)	0.0557 (13)	0.0322 (9)	0.0028 (9)	0.0092 (8)	−0.0079 (8)
N3	0.0471 (9)	0.0669 (14)	0.0292 (9)	0.0081 (10)	0.0030 (7)	0.0083 (9)
C1	0.0261 (7)	0.0224 (9)	0.0276 (9)	−0.0019 (7)	0.0039 (6)	0.0026 (7)
C2	0.0316 (8)	0.0280 (10)	0.0245 (9)	−0.0024 (8)	0.0057 (7)	0.0030 (7)
C3	0.0320 (8)	0.0307 (10)	0.0333 (10)	−0.0019 (8)	0.0112 (7)	−0.0015 (8)
C4	0.0298 (8)	0.0293 (10)	0.0354 (10)	0.0036 (8)	0.0074 (7)	0.0038 (7)
C5	0.0289 (8)	0.0309 (11)	0.0278 (10)	−0.0009 (7)	0.0031 (7)	0.0048 (7)
C6	0.0284 (8)	0.0250 (9)	0.0247 (9)	−0.0028 (7)	0.0046 (6)	0.0009 (7)
C7	0.0282 (8)	0.0257 (9)	0.0261 (9)	−0.0028 (7)	0.0049 (7)	0.0018 (7)
C8	0.0263 (8)	0.0293 (10)	0.0275 (9)	−0.0007 (7)	0.0048 (6)	0.0011 (7)
C9	0.0312 (8)	0.0310 (10)	0.0260 (9)	−0.0016 (8)	0.0053 (7)	0.0011 (7)
C10	0.0363 (9)	0.0413 (12)	0.0239 (9)	−0.0014 (9)	0.0050 (7)	−0.0023 (8)
C11	0.0372 (9)	0.0448 (12)	0.0228 (9)	−0.0028 (9)	0.0068 (7)	−0.0015 (8)
C12	0.0331 (8)	0.0265 (10)	0.0236 (9)	0.0008 (8)	−0.0009 (7)	0.0061 (7)
C13	0.0306 (8)	0.0300 (10)	0.0240 (9)	0.0026 (8)	0.0004 (7)	0.0012 (7)
C14	0.0331 (9)	0.0307 (10)	0.0271 (10)	0.0029 (8)	0.0024 (7)	−0.0004 (8)
C15	0.0314 (8)	0.0331 (11)	0.0285 (10)	0.0005 (8)	0.0025 (7)	−0.0026 (8)
C16	0.0334 (9)	0.0348 (11)	0.0296 (10)	0.0004 (8)	0.0046 (7)	−0.0037 (8)
C17	0.0336 (9)	0.0337 (11)	0.0283 (10)	0.0007 (8)	0.0037 (7)	−0.0009 (8)
C18	0.0368 (9)	0.0378 (12)	0.0305 (10)	0.0005 (9)	0.0074 (8)	−0.0024 (8)
C19	0.0342 (9)	0.0357 (12)	0.0306 (10)	−0.0003 (8)	0.0064 (7)	−0.0008 (8)
C20	0.0403 (10)	0.0395 (13)	0.0417 (12)	−0.0015 (10)	0.0130 (9)	−0.0025 (9)
C21	0.0388 (11)	0.0439 (14)	0.0613 (16)	−0.0052 (10)	0.0133 (10)	−0.0039 (12)

### Geometric parameters (Å, º)

**Table:**

O1—C8	1.214 (2)	C13—H13B	1.02 (2)
N1—C8	1.372 (2)	C14—C15	1.525 (3)
N1—C1	1.406 (2)	C14—H14A	1.05 (2)
N1—C12	1.461 (2)	C14—H14B	1.00 (2)
N2—C10	1.147 (3)	C15—C16	1.521 (3)
N3—C11	1.142 (3)	C15—H15A	1.01 (2)
C1—C2	1.377 (3)	C15—H15B	1.04 (2)
C1—C6	1.410 (2)	C16—C17	1.526 (3)
C2—C3	1.396 (3)	C16—H16A	1.02 (3)
C2—H2	0.98 (2)	C16—H16B	1.01 (2)
C3—C4	1.386 (3)	C17—C18	1.519 (3)
C3—H3	0.99 (2)	C17—H17A	1.02 (3)
C4—C5	1.389 (3)	C17—H17B	1.01 (2)
C4—H4	0.94 (2)	C18—C19	1.524 (3)
C5—C6	1.393 (3)	C18—H18A	1.00 (3)
C5—H5	0.96 (2)	C18—H18B	1.01 (3)
C6—C7	1.440 (2)	C19—C20	1.518 (3)
C7—C9	1.350 (3)	C19—H19A	1.03 (3)
C7—C8	1.520 (2)	C19—H19B	1.04 (2)
C9—C10	1.437 (3)	C20—C21	1.522 (3)
C9—C11	1.444 (3)	C20—H20A	1.02 (3)
C12—C13	1.526 (2)	C20—H20B	1.01 (3)
C12—H12A	1.06 (2)	C21—H21A	1.01 (3)
C12—H12B	1.01 (2)	C21—H21B	0.97 (3)
C13—C14	1.525 (3)	C21—H21C	1.00 (3)
C13—H13A	1.02 (2)		
			
C8—N1—C1	110.66 (14)	C15—C14—H14A	109.4 (13)
C8—N1—C12	123.47 (15)	C13—C14—H14B	110.6 (12)
C1—N1—C12	125.69 (15)	C15—C14—H14B	109.3 (13)
C2—C1—N1	128.09 (16)	H14A—C14—H14B	105.7 (17)
C2—C1—C6	121.89 (16)	C16—C15—C14	114.37 (16)
N1—C1—C6	109.99 (15)	C16—C15—H15A	107.9 (13)
C1—C2—C3	117.34 (17)	C14—C15—H15A	109.6 (13)
C1—C2—H2	121.3 (12)	C16—C15—H15B	109.9 (12)
C3—C2—H2	121.3 (12)	C14—C15—H15B	110.8 (12)
C4—C3—C2	121.69 (18)	H15A—C15—H15B	103.6 (18)
C4—C3—H3	119.3 (13)	C15—C16—C17	113.24 (16)
C2—C3—H3	119.0 (13)	C15—C16—H16A	109.4 (13)
C3—C4—C5	120.72 (18)	C17—C16—H16A	109.8 (14)
C3—C4—H4	122.2 (13)	C15—C16—H16B	110.9 (13)
C5—C4—H4	117.1 (13)	C17—C16—H16B	108.5 (13)
C4—C5—C6	118.63 (17)	H16A—C16—H16B	104.6 (18)
C4—C5—H5	119.9 (13)	C18—C17—C16	113.37 (16)
C6—C5—H5	121.4 (13)	C18—C17—H17A	110.1 (14)
C5—C6—C1	119.70 (17)	C16—C17—H17A	108.2 (14)
C5—C6—C7	133.42 (17)	C18—C17—H17B	109.1 (13)
C1—C6—C7	106.87 (15)	C16—C17—H17B	108.2 (13)
C9—C7—C6	131.53 (16)	H17A—C17—H17B	107.6 (19)
C9—C7—C8	121.89 (16)	C17—C18—C19	114.73 (17)
C6—C7—C8	106.55 (15)	C17—C18—H18A	108.3 (14)
O1—C8—N1	127.13 (16)	C19—C18—H18A	109.8 (14)
O1—C8—C7	126.96 (17)	C17—C18—H18B	109.1 (14)
N1—C8—C7	105.90 (15)	C19—C18—H18B	108.1 (15)
C7—C9—C10	124.25 (16)	H18A—C18—H18B	106 (2)
C7—C9—C11	121.24 (18)	C20—C19—C18	113.30 (17)
C10—C9—C11	114.51 (16)	C20—C19—H19A	109.3 (13)
N2—C10—C9	173.7 (2)	C18—C19—H19A	110.1 (14)
N3—C11—C9	179.3 (3)	C20—C19—H19B	112.3 (13)
N1—C12—C13	113.70 (15)	C18—C19—H19B	107.9 (12)
N1—C12—H12A	106.2 (12)	H19A—C19—H19B	103.6 (18)
C13—C12—H12A	108.8 (11)	C19—C20—C21	113.11 (19)
N1—C12—H12B	106.6 (12)	C19—C20—H20A	109.7 (14)
C13—C12—H12B	110.0 (12)	C21—C20—H20A	110.3 (14)
H12A—C12—H12B	111.6 (17)	C19—C20—H20B	106.0 (13)
C14—C13—C12	114.63 (16)	C21—C20—H20B	110.7 (14)
C14—C13—H13A	108.8 (12)	H20A—C20—H20B	107 (2)
C12—C13—H13A	105.0 (13)	C20—C21—H21A	110.0 (15)
C14—C13—H13B	112.0 (12)	C20—C21—H21B	112.0 (18)
C12—C13—H13B	108.1 (11)	H21A—C21—H21B	108 (2)
H13A—C13—H13B	108.0 (17)	C20—C21—H21C	109.0 (16)
C13—C14—C15	111.49 (16)	H21A—C21—H21C	110 (2)
C13—C14—H14A	110.3 (13)	H21B—C21—H21C	107 (2)
			
C8—N1—C1—C2	176.31 (17)	C1—N1—C8—C7	1.67 (19)
C12—N1—C1—C2	1.2 (3)	C12—N1—C8—C7	176.95 (15)
C8—N1—C1—C6	−1.9 (2)	C9—C7—C8—O1	−0.6 (3)
C12—N1—C1—C6	−177.06 (16)	C6—C7—C8—O1	177.92 (18)
N1—C1—C2—C3	179.73 (17)	C9—C7—C8—N1	−179.39 (17)
C6—C1—C2—C3	−2.3 (3)	C6—C7—C8—N1	−0.88 (19)
C1—C2—C3—C4	1.1 (3)	C6—C7—C9—C10	−177.85 (18)
C2—C3—C4—C5	0.7 (3)	C8—C7—C9—C10	0.2 (3)
C3—C4—C5—C6	−1.4 (3)	C6—C7—C9—C11	1.6 (3)
C4—C5—C6—C1	0.3 (3)	C8—C7—C9—C11	179.71 (17)
C4—C5—C6—C7	178.57 (19)	C8—N1—C12—C13	112.3 (2)
C2—C1—C6—C5	1.6 (3)	C1—N1—C12—C13	−73.1 (2)
N1—C1—C6—C5	179.93 (15)	N1—C12—C13—C14	−62.1 (2)
C2—C1—C6—C7	−177.09 (16)	C12—C13—C14—C15	−173.98 (15)
N1—C1—C6—C7	1.25 (19)	C13—C14—C15—C16	179.80 (16)
C5—C6—C7—C9	−0.3 (3)	C14—C15—C16—C17	−178.34 (16)
C1—C6—C7—C9	178.09 (19)	C15—C16—C17—C18	−179.93 (17)
C5—C6—C7—C8	−178.65 (19)	C16—C17—C18—C19	−179.68 (17)
C1—C6—C7—C8	−0.23 (18)	C17—C18—C19—C20	−179.44 (18)
C1—N1—C8—O1	−177.13 (18)	C18—C19—C20—C21	178.45 (19)
C12—N1—C8—O1	−1.8 (3)		
